# A New Perspective on Alzheimer's Risk: Index Development With ADNI Data

**DOI:** 10.1002/alz70860_096872

**Published:** 2025-12-23

**Authors:** Tahera Ahmed, Kuldeep Kumar, Ping Zhang

**Affiliations:** ^1^ Bond University, Gold Coast, QLD, Australia; ^2^ Kids Research Institute, Perth, Western Australia, Australia; ^3^ Griffith University, Brisbane, QLD, Australia

## Abstract

**Background:**

Alzheimer's disease (AD) is a prevalent neurodegenerative condition impacting the elderly population. Despite its widespread occurrence, the precise etiological factors remain elusive, emphasizing the critical need for early detection and intervention to mitigate disease progression. This study aims to development of an Alzheimer's Risk Index, a novel tool designed to evaluate socio‐demographic factors, lifestyle, medical history, behavioural patterns, and neuropsychological factors contributing to Alzheimer's risk and cognitive decline.

**Method:**

In this study, we used participants aged 55 to 91 from the ADNI database and employed a unique method using Factor Analysis (FA) in the index development

**Result:**

This study's cutting‐edge approach to index development using ADNI database and PCA provides valuable insights into potential associations between the Alzheimer's Risk Index and the prevalence of AD.

**Conclusion:**

This index is expected to be easily accessible to the general population as a guidance for seeking medical diagnosis of AD early detection.

